# Developing Guidelines for Conducting Stigma Research With Transgender and Nonbinary Individuals: Protocol for Creation of a Trauma-Informed Approach to Research

**DOI:** 10.2196/66800

**Published:** 2025-01-06

**Authors:** Augustus Klein, Sarit A Golub, Danielle Berke, Elijah Castle

**Affiliations:** 1 Department of Psychology Hunter College of the City University of New York (CUNY) New York, NY United States; 2 Hunter Alliance for Research and Translation Hunter College of the City University of New York (CUNY) New York, NY United States; 3 Basic and Applied Social Psychology Department of Psychology The Graduate Center of the City University of New York (CUNY) New York, NY United States

**Keywords:** transgender, non-binary, HIV prevention and treatment, stigma research, trauma-informed

## Abstract

**Background:**

Transgender and nonbinary individuals have received increasing attention within HIV research, with studies documenting the pervasive role stigma plays in creating and sustaining health inequities. However, the proliferation of HIV stigma research with this population has also raised concerns about research practices that may unintentionally stigmatize or retraumatize the very communities they are designed to benefit. Conducting stigma research is critical for generating accurate information about HIV epidemiology, risk and protective factors, and intervention strategies for transgender and nonbinary individuals. Yet, little research has directly examined the experiences of transgender and nonbinary individuals when participating in these studies or identified specific research practices (eg, recruitment materials or study framing, choice of specific survey measures, data collection protocols, and researcher behaviors) that may influence study participation, retention, and data quality. Equally important, research has not adequately examined the potential for unintended harm due to emotional distress experienced by participating in such research and what specific strategies might mitigate against potential distressful research experiences.

**Objective:**

This study aimed to develop a set of empirically based trauma-informed guidelines for conducting HIV-related stigma research with transgender and nonbinary individuals to increase researchers’ capacity to recruit and retain transgender and nonbinary individuals in HIV-related stigma research, enhance the quality of data collected, and reduce unintentional harm in stigma research methodology.

**Methods:**

The study will engage in primary data collection using both qualitative and quantitative methodology. First, we will use in-depth qualitative interviews with 60 participants representing 3 participant groups: researchers, mental health clinicians, and transgender and nonbinary individuals who have participated in HIV-related and sexual health research. Second, the qualitative findings will be used to develop an initial set of survey items representing a preliminary set of guidelines. Third, we will engage 75 participants in a 3-round modified Delphi method, to refine the guidelines and promote their acceptability among key stakeholders.

**Results:**

The study is funded by the National Institute of Mental Health starting in July 2022 and data collection began January 2023. The study’s findings underscore the critical importance of adopting a trauma-informed approach to HIV stigma research with transgender and nonbinary individuals.

**Conclusions:**

To make meaningful strides in stigma research, it is imperative to examine experiences of stigma that may happen within the research context and identify strategies for improving data quality and reducing unintentional harm in study recruitment, methodology, implementation, and dissemination.

**International Registered Report Identifier (IRRID):**

DERR1-10.2196/66800

## Introduction

### Background

Over the past 2 decades, transgender individuals, particularly Black and Latina transgender women, have received increasing attention within HIV research [1-9]. Such research has consistently documented the pervasive role stigma plays in creating and sustaining health inequities among this population. However, this research proliferation has also raised questions about practices that may unintentionally stigmatize or retraumatize the very communities they are designed to benefit [4,10-15]. One area of particular concern is the measurement of stigma as part of HIV-related research with transgender and nonbinary individuals. First, there is concern that specific items within commonly used measures may unintentionally recreate or activate stigma. For example, many stigma and minority stress scales [16-19] include items that may be experienced as stigmatizing (eg, “Being transgender is disgusting to me”) [19] or ask participants to recount and relive traumatic experiences (eg, history of physical and sexual violence, family rejection, and experiences of discrimination and harassment) to document their association with negative affect or health behavior [2]. There has been considerable debate within the larger field of trauma-related research as to whether recalling and answering questions about past trauma has negative or positive consequences for study participants [20-29]. However, there is surprisingly limited research within HIV science on the potential emotional impacts of participating in stigma research. Second, there are no evidence-based guidelines for person-centered, trauma-informed, and actively destigmatizing implementation of HIV stigma research. Conducting stigma research is critical for generating accurate information about HIV epidemiology, risk and protective factors, and intervention strategies for transgender and nonbinary individuals. Yet little research has directly examined the experiences of transgender and nonbinary individuals when participating in these studies, or identified specific research practices (eg, recruitment materials or study framing, choice of specific survey measures, data collection protocols, and researcher behaviors) that may influence study participation, retention, and data quality [30]. Equally important, research has not adequately examined the potential for unintended harm due to emotional distress experienced by participating in such research and what specific strategies might mitigate against potential distressful research experiences.

### Impact of Stigma on HIV-Related Outcomes

Research documenting multiple and intersecting structural factors, including racism, sexism, transphobia, homophobia, and other systems of stigmatization, oppression, and traumatic victimization that contribute to the disproportionately high rates of HIV infection and HIV-related morbidity in this population [2-4,6,7,14,31-35]. One of the strengths of HIV research in this area has been its intersectional focus, [36] as well as the acknowledgement that stigma operates at individual, interpersonal, and structural levels. Intersectional approaches demonstrate the ways in which HIV-stigma and other sources of stigma occur simultaneously and interact to impact transgender and nonbinary individual’s daily experience, health outcomes, and engagement with care. Research suggests that transgender and nonbinary individuals may experience this impact as traumatic, and support trauma-informed approaches that seek to mitigate retraumatization within the provision of care [37-43]. Transgender and nonbinary individuals health disparities have been directly linked to intersectional and multidimensional stigma processes [37,41,44,45], underscoring the extent to which this approach is essential for advancements in transgender and nonbinary individual–specific stigma frameworks, measurement, and intervention development.

### Potential for Unintentional Harm

Within the fields of clinical psychology and neuroscience, there has been intense debate about the impact of research about traumatic experiences on study participants [20-29]. Some evidence suggests that such research might lead to traumatization or cause further harm to those with previous exposure to violence or abuse. One meta-analysis of studies about traumatic events found that approximately 25% (IQR 4.3%-50%) of adult participants reported distressing impacts (ie, unexpected upset, negative emotions, unwanted thoughts, or distress) as a result of research participation. Although, most people find that participating in trauma research is distressing, they also report that they find it worthwhile and meaningful [28]. Neuroscience research on memory reconsolidation suggests that the context and content of memory reactivation may determine its harmful or therapeutic impact. Data indicate that research participants anticipate or attribute negative impacts to study participation; in 1 large-scale study of traumatic experience, 94% of participants rated their participation as more than minimal risk, with participants that had greater previous exposure to trauma reporting higher levels of distress [46]. However, limited research within the field of HIV has directly examined the potential impact of stigma research itself on transgender and nonbinary participants.

### Impact on Engagement in HIV Prevention and Care

Growing recognition of the importance of community partnership for HIV research with transgender and nonbinary individuals has led to a rise of studies that rely on community health centers or other service agencies for research recruitment and implementation [47-50]. It is well established that experiences of stigma within these settings may negatively impact transgender and nonbinary individual’s willingness to receive needed health care, including HIV testing, treatment, or pre-exposure prophylaxis [37-39,51,52]. If stigmatizing or harmful research is conducted within these settings, there is the potential to reduce transgender and nonbinary individual’s trust in the very organizations upon which they depend to access life-saving services. As such, it is essential to understand whether and under what conditions HIV stigma research may result in unintentional harm or stigmatization of transgender and nonbinary individuals, and how harmful impacts can be reduced.

### Impact on Recruitment, Engagement, and Data Quality

Evidence indicates that mistrust of research projects, study teams, or settings leads to difficulties with recruitment and retention of study participants and may be associated with false or misleading responses to study measures. Studies indicate that research mistrust is particularly strong among transgender and nonbinary participants, who may report feeling used or mistreated within HIV research contexts [53-55]. In 1 study, transgender women reported that research scripts and procedures can be experienced as microaggressions [55]. Critical HIV-related stigma research is likely to underperform and fail to provide much needed data on stigma processes if participants are discouraged from participating or alienated from the research enterprise by perceptions that stigma research is itself stigmatizing [56].

### Need for Empirically Grounded Research Guidelines

Many HIV stigma studies report incorporating intentionally affirming components into their research practice, such as measures of self-esteem, community-connectedness, or other resilience factors [57,58]. However, there has been no systematic compilation of these strategies, or analysis of their potential use in reducing stigma experiences. This research gap results in a lack of consensus of what is meant by trauma-informed HIV stigma research, as well as gaps in how to best implement and evaluate stigma-reducing measures in HIV research practice. Outside of HIV research, there are models for patient-centered, trauma-informed health research [59] that could be adapted to better inform implementation strategies, but such adaptation needs to be grounded in empirical data from participants, researchers, and practitioners with direct experience in the field.

### Theoretical Frameworks

This study is based on an interdisciplinary integration of minority-stress and trauma-informed theoretical frameworks to explain health inequity. Minority stress theory [60] is a strong epidemiological framework for explaining disparity but is less precise in specifying mechanisms of an individual stigmatized person’s behavior, feelings, or experience. To complement this theory, we incorporate a conceptual framework developed specifically to support the mental health of TGNB individuals [61], which combines 2 trauma-informed care models to guide our research questions and analysis. The first, developed by Fallot & Harris [62,63], emphasizes five principles of interaction: safety, trustworthiness, collaboration, choice, and empowerment. The second, guiding principles of trauma-informed care created by the Substance Abuse and Mental Health Services Administration within the US Department of Health and Human Services, focuses on attention to cultural, historical, and gender issues that impact power relationships, privilege, and oppression [64]. This trauma-informed theoretical approach attends to the potential presence of trauma-related symptoms on the thoughts, feelings, needs, and reactions of TGNB research participants, and aims to actively disrupt retraumatization through the creation of interpersonal processes and settings that emphasize safety, trust, collaboration, empowerment, and choice. Rather than focusing on isolating and describing stigmatizing internal and external experiences that happen to a marginalized person, a trauma-informed approach to stigma research centers how those marginalizing experiences thwart a survivor’s wellness, healing, and resilience [65]. Trauma-informed approaches are, therefore, inherently person-centered, and strengths-based in that they entail recognition of the signs and symptoms of trauma in an individual’s behavior and guide clinical responses to minimize negative impact on the survivor’s natural recovery process [64]. These characteristics are likely to support greater engagement, quality, and benefit of HIV research among transgender and nonbinary individuals [55].

### Study Aims

The primary aim of this study is to develop empirically informed, trauma-informed guidelines for conducting HIV-related stigma research with transgender and nonbinary individuals. This work seeks to address critical gaps in the field by improving recruitment, retention, and data quality while reducing the potential for harm during research participation. Specifically, the study focuses on the following aims. First, this study aims at understanding participant experiences by conducting in-depth interviews with transgender and nonbinary participants who have engaged in HIV or sexual health-related research to explore their experiences, including factors that contribute to or mitigate distress, stigma, and harm. This aim seeks to uncover the nuanced challenges and opportunities for designing research that supports participant well-being. Second, this study aims to gather stakeholder perspectives by conducting in-depth interviews with researchers and mental health clinicians to identify current practices, challenges, and strategies for implementing trauma-informed and person-centered approaches in HIV stigma research. This aim emphasizes understanding the perspectives of professionals who interact with transgender and nonbinary populations in research and clinical settings. Third, this study aims to develop and refine trauma-informed guidelines by using findings from the first and second aims to create a preliminary set of trauma-informed research guidelines. These guidelines will be refined through a modified Delphi process involving transgender and nonbinary individuals, researchers, and mental health professionals to ensure their relevance, acceptability, and applicability across diverse research contexts.

By integrating qualitative interviews with a structured consensus-building approach, this study aims to establish actionable and evidence-based recommendations. These guidelines will enhance the ethical rigor and methodological quality of HIV stigma research, contributing to improved health equity and reducing unintentional harm in research practices.

## Methods

### Study Objectives

Our study is designed to address a critical gap in existing HIV stigma research with transgender and nonbinary individuals by examining experiences of stigma within the research context and identifying strategies for improving data quality and reducing unintentional harm in study recruitment, methodology, implementation, and dissemination. This study will engage in primary data collection using both qualitative and quantitative data collection methodology. To accomplish the study aims we will first, conduct in-depth interviews with transgender and nonbinary individuals (n=30) to better understand how they understand and experience participation in HIV-related stigma research, including willingness to respond to questions about stigma and factors that may contribute to or mitigate potential distressing or stigmatizing experiences within the research context. Second we will conduct in-depth interviews with 2 groups: investigators who conduct HIV-related stigma research with transgender and nonbinary individuals, to better understand perceptions of and experiences with conducting stigma research with transgender and nonbinary individuals and compile existing strategies for mitigating harm (n=15) and mental health professionals (n=15) who provide care to transgender and nonbinary individuals to better understand ways in which experiences of stigma can be measured and studies can be conducted in a manner that is person-centered, trauma-informed, and actively destigmatizing. Third, we will develop a set of empirically informed guidelines for conducting HIV stigma research with transgender and nonbinary individuals. We will use a modified Delphi technique to engage a panel of transgender and nonbinary individuals, mental health providers, and researchers in a consensus building process to identify practical recommendations for person-centered, trauma-informed recruitment, measurement, and conduct of HIV-related stigma research with transgender and nonbinary individuals.

The sample sizes for this study were designed to ensure robust data collection and meaningful analysis across all phases. For aims 1 and 2, the sample size was chosen based on recommendations for similar qualitative inquires to ensure thematic saturation and demographic diversity [66-69]. In aim 3 the Delphi panel will consist of 75 participants (25 transgender and nonbinary individuals, 25 researchers, and 25 clinicians), aligning with best practices for achieving reliable consensus and providing robustness against attrition [68,70,71]. These sample sizes ensured methodological rigor, demographic representativeness, and the capacity to develop empirically informed, trauma-informed research guidelines.

### Qualitative Interviews

#### Aim 1: Semistructured Qualitative Interviews With Transgender and Nonbinary Individuals

##### Overview

In aim 1, we will conduct a series of in-depth semistructured qualitative interviews with 30 transgender and nonbinary individuals.

##### Participants

Our sample will be stratified by gender identity and HIV status. We will ensure that >40% of participants will be aged 18-29 years and at least 70% people of color, due to the disproportionately high HIV infection rates among these populations and lack of access to HIV prevention and treatment services. Eligible participants will be aged >18 years; identify as transgender, nonbinary, or gender diverse; and have participated in an HIV prevention or treatment related research study.

##### Recruitment

Participants will be recruited through existing research panels, transgender health-related social media and listservs, and word of mouth. Interested participants will fill out an eligibility screener survey. For those who are deemed eligible, they will be provided a link to the study website [72] to schedule their interview at a day and time of their choice, with the study team member of their choice. Participants will be provided with a link to an electronic consent form on the study website, as well as the interview guide before the interview. The interviewer will review the consent form with the participant and obtain verbal consent before conducting the interview.

##### Interview Procedures

Interviews will be designed to last no longer than an hour and a half, and participants will be compensated US $80 for their time. All interviews will contain a core set of questions to assess how transgender and nonbinary individuals understand and experience participation in HIV-related stigma research, including decision-making processes around participation, perceptions of study purpose, risks and benefits, participants’ willingness to respond to questions on stigma, factors that may contribute to or mitigate potential distressful or stigmatizing experiences within the research context, and opinions of the research after participation. Participants will also be asked to identify specific strategies to enhance researcher’s capacity to design and implement stigma research that is person-centered, trauma-informed, and actively destigmatizing.

#### Aim 2: Semistructured Qualitative Interviews With Researchers and Mental Health Professionals

##### Overview

In aim 2, we will conduct a series of in-depth semistructured qualitative interviews with 2 groups, investigators who conduct HIV-related stigma research with transgender and nonbinary individuals (n=15) and mental health providers who provide care to transgender and nonbinary adults (n=15).

##### Participants

Investigators (n=15) will be stratified by career stage: early (n=5), middle (n=5), or late (n=5) stage investigators to represent a range of perspectives and experiences with conducting stigma research with transgender and nonbinary individuals. Our sample of mental health professionals (n=15) will include mental health clinicians with a master’s degree in either social work, marriage and family therapy, or mental health counseling or a doctoral degree in clinical psychology (PhD or PsyD) who provide individual or group therapy to transgender and nonbinary adults (aged 18 years or older) and have training in providing trauma-specific or trauma-informed clinical practice.

##### Recruitment

Early, middle, or late-stage investigators with existing HIV-related transgender health research will be identified by Google Scholar and the National Institutes of Health (NIH) RePORTER. Investigators will be contacted by a study team member by email and invited to participate in our study. Mental health professionals will be recruited through professional networks and word of mouth. Interested participants will fill out an eligibility screener survey. For those who are deemed eligible, they will be contacted by a study team member to schedule their interview. Participants will be provided with a copy of the study consent form. The interviewer will review the consent form with the participant and obtain verbal consent before conducting the interview.

##### Interview Procedures

Aim 2 will consist of 30 semistructured interviews with 2 groups, investigators who conduct HIV-related stigma research with transgender and nonbinary and mental health clinicians who provide counseling to transgender and nonbinary adults (aged 18 years or older). Interviews are designed to last no more than an hour and a half, and participants will be compensated US $50 for their time. *Interviews with investigators* will assess understanding experiences of conducting HIV-related stigma research with transgender and nonbinary individuals, including perceptions of study purpose, risks and benefits, question and measurement selection and creation, and factors that may have contributed to or mitigated against distressful or stigmatizing experiences within the research context. *Interviews with mental health professionals* will focus on identifying ways in which experiences of stigma can be measured in a manner that is person-centered, trauma-informed, and actively destigmatizing, including suggestions for using person-centered, trauma-informed language when developing questions or measures, and strategies to assist researchers around developing protocols and procedures to assess for and address potential distressful situations within the research context.

### Development of Interview Guide (Aims 1 and 2)

The interview guide was developed through a collaborative, trauma-informed approach to ensure that all protocols and procedures reflected the study’s goals and minimized potential harm to participants. This process involved a 3-step methodology with the transgender-identified research team. First, the team conducted an in-depth review of existing trauma-informed care models and adopted the framework developed by The Institute on Trauma and Trauma-Informed Care (ITTC) at the University of Buffalo, which emphasizes the principles of safety, choice, empowerment, collaboration, and trustworthiness. Second, components from ITTC’s Trauma-Informed Organizational Change Manual [73] were adapted and integrated into the research procedures, including participant recruitment, consent, interview data collection, and analysis, to align all research activities with a trauma-informed approach. Third, the interview questions, prompts, and scripts were mapped to the ITTC framework to minimize risks of retraumatization and ensure a supportive environment for participants. Interview questions were refined to align with the study’s 4 overarching research questions and tailored to the perspectives of the 3 participant groups, researchers, community stakeholders, and mental health clinicians, ensuring comparability and analytic integrity across the study phases (Multimedia Appendix 1).

### Qualitative Analysis Plan (Aims 1 and 2)

Data collected in aims 1 and 2 will be analyzed using rapid qualitative analytic methods, [74-77] to identify key themes that best reflect the research decision-making process, perceptions and experiences of transgender and nonbinary individuals. We will assign four trained team members to summarize a subset of the interview transcripts independently, extracting key data into a summary template based on our framework and interview guide; triangulate key themes in the transcript through documenting observations, quotations, and reflections into the summary template; meet to compare and combine templates for each interview; and create a comprehensive matrix identifying common themes and contrasts across and within stakeholder groups.

### Application of Knowledge Gained in Aims 1 and 2 to Aim 3

In preparation for aim 3, we will use the themes that were identified in the qualitative interviews to develop a preliminary list of survey items for use in the first stage of the modified Delphi process (aim 3) [78]. The use of qualitative interviews in a pre-Delphi phase [78-80] allows for all relevant stakeholder groups to guide the Delphi process by contributing to the development of the first round Delphi survey. To ensure that the Delphi survey items describe and capture the perceptions and experiences of all the stakeholder groups participating in aim 3, we will develop items using the language and narratives participants use during their interviews [78,80,81]. For example, the language used to describe and the meaning behind core concepts within stigma research may differ distinctly between stakeholder groups and could contribute to inaccurate measurement and interpretation of research findings. By integrating language from each stakeholder group into the Delphi survey we hope to better reflect the experiences of all relevant stakeholders involved in this process [79,82,83]. We will then compare the themes and associations derived from Aims 1 and 2 to better understand areas of concordance and discordance between stakeholder groups [78-81,84]. For example, it is possible that interview findings highlight similar domains across participant groups that are key to understanding and improving stigma research procedures, yet what is important to address within these domains may differ by stakeholder group. The use of qualitative interview findings will help further parse out and include survey items that address this complexity.

### Aim 3: Development of Empirically Informed Guidelines for Conducting Stigma Research With Transgender and Nonbinary Individuals

#### Overview

To accomplish aim 3 we will use a 3-round modified Delphi method [85] to develop a set of empirically informed guidelines for conducting HIV-related stigma research with transgender and nonbinary individuals. The modified Delphi method will include 4 structured steps: panel formation, quantitative survey development, data collection and analysis, and guideline development [68,69,85,86].

#### Panel Formation

We will recruit 75 participants from the following 3 stakeholder groups, transgender and nonbinary individuals (n=25), researchers (n=25), and mental health professionals (n=25). Our sample size is based on previous studies which demonstrate that this sample size provides stable results robust to participant attrition or inconsistent responding [68,70,71]. Individuals who participated in aims 1 and 2 will be recruited to participate in aim 3.

#### Survey Development

Survey development will occur in 3 steps. First, the transcripts and codes from aims 1 and 2 will be reviewed to draft the initial survey items based on the most salient themes and domains pertinent to all aspects of the research process, including recruitment, measurement, retention, and dissemination. Each survey item will be designed to capture a single idea and be easily understood by participants. Second, we will develop a rating scale for the Delphi survey items that best capture the original content from aims 1 and 2. Third, we will finalize the items and organize the survey into the following 3 sections, Trauma-Informed Principles for Research Practice, Trauma-Informed Research Standards, and Trauma-Informed Research Competencies.

#### Quantitative Data Collection and Analysis

A survey will be administered online by REDCap (Research Electronic Data Capture) [87,88] at 3 time points (rounds 1-3). Participants will be compensated US $40 for their time after the completion of each survey. In each round, participants will be asked to rate each item on a 5-point scale indicating whether the item should be included in the research guidelines. Survey items will be categorized according to the decision matrix in Textbox 1, which has been widely used in previous studies [85,86]. In round 1, all items created by our study team will be included in the survey. At the end of each survey section in round 1, participants will be encouraged to provide feedback on items (ie, how to improve wording or messaging) and suggest novel items to include [68,85,86]. After round 1, we will analyze survey responses, including participant comments, and edit existing or draft new items to be included in the round 2 survey based on participant suggestions. In the second-round survey, participants will rerate items that receive a near miss in the first round and rate new items the research team crafted based on suggestions from participants in round 1. At the end of rounds 1 and 2, participants will be sent a report outlining survey results. Statements to be rerated will be displayed with the overall percentages for each rating and then by stakeholder group so participants can compare their response with others’ responses. The report will allow participants to consider whether to retain or change their ratings in the next round. Finally, in round 3, only items rated for the first time in round 2 or received a near miss rating will be included in the final survey (Textbox 1).

**Decision matrix for developing expert consensus.**

**Endorsed**

- If between 80%-100% of participants of each group rate a statement as either essential or important, it will be endorsed as a guideline.

**Re-rated (near miss)**

- If between 80%-100% of participants of each group rate a statement as either essential or important, it will be endorsed as a guideline.

**Rejected**

- If none of the above conditions are met, a statement will be rejected for inclusion as a guideline.

#### Guideline Development

At the end of round 3, the research team will create a document comprised of the endorsed items to be widely disseminated. Once the document is completed, participants will be invited to attend a 1-day virtual convening where we will present and discuss the final guidelines document as well as the goals and next steps for dissemination and implementation.

### Ethical Considerations

#### Ethics Approval

The study received expedited approval from the City University of New York Human Research Protection Program (#2022-0280-Hunter).

#### Informed Consent

Participants were electronically sent a consent form and interview guide in advance to review at their own pace once their interview was scheduled. Before the start of each interview a trained study team member reviewed the consent form with participants and obtained verbal consent to participate. Before completing the Delphi survey participants affirmed that the agreed to participate.

#### Privacy and Confidentiality

All data were anonymized to protect participant identities.

#### Compensation

Participants were compensated US $40- US $80, depending on the study phase, to ensure fairness.

## Results

The study was funded by the National Institute of Mental Health from July 1, 2022, to June 30, 2024. The study successfully achieved its specific aims. We successfully completed data collection and analysis for aims 1 and 2 recruiting 30 transgender and nonbinary participants (aim 1) and a total of 34 participants in aim 2 (17 researchers and 17 clinicians). As outlined above, we directly applied the knowledge gained to the development of the preliminary set of guidelines voted on in aim 3. The Delphi process for aim 3 is entering its final survey round, and we are actively preparing the finalized guidelines for dissemination (Table 1).

**Table 1:** Study timeline and progress by aims.

Phase	Planned timeline	Current progress
Aim 1: interviews	January 2023-June 2023	Completed (30 participants)
Aim 2: interviews	July 2023-December 2023	Completed (34 participants)
Application of knowledge gained from aims 1 and 2	January 2024-March 2024	Completed
Aim 3: Delphi survey	March 2024-June 2024	Ongoing (final survey round)

## Discussion

### Principal Findings

This study addresses a critical gap in HIV-related stigma research by developing trauma-informed guidelines to improve the quality of research involving transgender and nonbinary individuals. HIV stigma research has historically documented the pervasive role of stigma in shaping health inequities but has largely overlooked the unintended harms participants may experience within the research process itself. By using an interdisciplinary, trauma-informed approach grounded in qualitative data collection and a modified Delphi method, this study emphasizes principles of safety, trustworthiness, collaboration, empowerment, and cultural sensitivity. The findings aim to enhance participant engagement, reduce emotional distress, and improve data quality, thereby advancing ethical and effective research practices. Through active collaboration with community members, researchers, and clinicians, this project provides a model for addressing stigma while centering participant well-being and resilience.

The study’s findings underscore the critical importance of adopting a trauma-informed approach to HIV stigma research with transgender and nonbinary individuals. Interviews with community members, researchers, and clinicians revealed the significant benefits of trauma-informed principles for enhancing participant trust, retention, and engagement while also improving the quality of collected data. Key findings include the following.

#### Higher Data Quality and Participant Engagement

Stakeholders across all groups emphasized that trauma-informed practices create safer and more supportive environments for participants, leading to more honest and comprehensive data. For instance, researchers noted that flexible interview formats and clear communication protocols increased participant willingness to share sensitive information.

#### Improved Team Dynamics and Research Outcomes

Researchers and clinicians reported that adopting trauma-informed principles, such as trustworthiness and collaboration, not only benefits participants but also enhances team cohesion and efficiency in implementing study protocols.

#### Ethical and Methodological Standards

The findings contributed to the development of trauma-informed research standards, including guidelines for ethical recruitment, harm minimization, and culturally sensitive data collection protocols. These standards prioritize participant autonomy and recognize the compounded effects of intersecting identities, such as race, gender, and sexual orientation​.

#### Institutional Support Needs

Participants consistently highlighted the necessity of institutional support for implementing trauma-informed practices. Recommendations include increased funding, researcher training, and revisions to institutional review board protocols to ensure systemic integration of these practices into research frameworks​.

#### Guidelines for Trauma-Informed Research

Preliminary guidelines developed through this study emphasize the integration of trauma-informed principles into every aspect of the research process. These include ensuring safety and dignity, using affirming language, and providing participants with opportunities for feedback and choice throughout their involvement in studies.

The findings collectively highlight the transformative potential of trauma-informed approaches for improving HIV stigma research practices. By addressing participant needs and mitigating harm, these practices ensure ethical rigor and enhance the impact of research on health equity for transgender and nonbinary populations.

#### Comparison to Previous Work

Previous research on HIV stigma has predominantly focused on documenting stigma and its health impacts without critically examining research practices themselves. Our study extends the NIH’s Stigma and Discrimination Toolkit [89] by addressing the need for guidelines to reduce stigma within the research context. This complements existing frameworks and fills a critical gap. By focusing on the process rather than outcomes alone, this research provides a novel contribution to the field.

#### Strengths and Limitations

The study’s strengths include a diverse, multidisciplinary research team with significant community representation, innovative use of the Delphi method, and the integration of trauma-informed principles. Limitations include the absence of a bioethicist on the team and potential challenges in generalizing findings to other populations beyond transgender and nonbinary individuals. Future research could explore the applicability of these guidelines in other contexts and populations.

#### Future Directions

Key next steps include developing researcher training modules and advocating for institutional review board and institutional policies that mandate trauma-informed practices. These guidelines could extend to other areas of health inequities research.

#### Dissemination Plan

This study prioritizes transparency and community engagement in disseminating findings. The dissemination plan aligns with trauma-informed principles by emphasizing accessibility, mutuality, and collaboration with the community and stakeholders.

#### Community-Focused Dissemination

A dedicated study website was created to provide participants and stakeholders with clear and accessible information about the study aims, process, and team. This platform has been instrumental in sharing updates, progress, and findings during the study period​.

Two newsletters were distributed to participants and posted on the website to update stakeholders about study progress and preliminary findings. These newsletters ensured continuous engagement with the community and maintained trust.​

Final study findings will be summarized in community-friendly formats, such as infographics and plain-language reports, and shared through newsletters and the website. This ensures that findings are accessible to diverse audiences, including those with varying levels of education and technical expertise.

#### Academic and Professional Dissemination

Findings have been shared at national and international conferences, ensuring visibility within academic and professional circles. Future presentations will focus on engaging institutional review boards, research institutions, and policy makers​.

The trauma-informed research guidelines will be published in academic journals and policy briefs, targeting audiences involved in HIV research, social work, and public health.

Workshops and webinars will be conducted for researchers and community health organizations to facilitate the implementation of the guidelines. These sessions will provide practical strategies for adopting trauma-informed practices.

#### Collaborative Dissemination

The final guidelines will be shared during virtual or in-person gatherings with study participants and community members. These sessions will include opportunities for participants to provide feedback and discuss the next steps for implementation.

Findings will be disseminated through partnerships with community-based organizations and health centers. This approach ensures that the guidelines reach the people and institutions directly involved in HIV stigma research and care.

#### Sustainability and Long-Term Impact

Recommendations for integrating trauma-informed practices into institutional review board review processes, funding applications, and researcher training will be disseminated to key institutions. This will include targeted briefings and resource-sharing with institutional review boards and funders to encourage systemic change​.

Training materials, including video tutorials and toolkits, will be developed to help researchers and institutions adopt the guidelines. These materials will be shared through academic and community networks. By using these dissemination strategies, this study aims to ensure that its findings are accessible, actionable, and impactful across diverse audiences, including researchers, policy makers, and the communities at the center of the research.

### Conclusion

To make meaningful strides in stigma research, it is imperative to examine experiences of stigma within the research context and identify strategies to improve data quality while reducing unintentional harm in study recruitment, methodology, implementation, and dissemination. This study addresses these critical needs by developing empirically informed trauma-informed guidelines for conducting HIV-related stigma research with transgender and nonbinary individuals.

Grounded in interdisciplinary theoretical frameworks and extensive collaboration with transgender and nonbinary participants, researchers, and clinicians, this study highlights the transformative potential of trauma-informed approaches. These guidelines emphasize the principles of safety, trustworthiness, collaboration, empowerment, and cultural sensitivity, demonstrating their capacity to enhance participant engagement, foster trust, and mitigate risks of retraumatization. Findings further underscore the importance of systemic institutional support, including training programs, funding mechanisms, and policy revisions at the institutional level, to ensure the widespread adoption of these practices.

The study achieved its goals by developing and refining these guidelines through rigorous qualitative analyses and a modified Delphi process. By embedding trauma-informed principles into research methodologies, this work sets a new standard for ethical and effective research practices. These guidelines have the potential to increase researchers’ capacity to recruit and retain transgender and nonbinary participants in stigma research, improve the quality of collected data, and reduce the unintended harms of research participation.

Next steps include broad dissemination of the guidelines through community-friendly formats, workshops, and academic publications. These efforts will ensure that the findings are accessible to a diverse range of stakeholders, including researchers, clinicians, policy makers, and community organizations. By reshaping HIV stigma research methodologies, this work contributes to advancing health equity and ethical research practices, with implications for other high-priority populations and fields of health disparity research.

## Appendix

Multimedia Appendix 1 Interview guide for Aims 1 and 2 by participant group.

Multimedia Appendix 2 Peer-reviewer report from HIV/AIDS Intra- and Inter-personal Determinants and Behavioral Interventions Study Section, Risk, Prevention and Health Behavior Integrated Review Group (HIBI) (National Institutes of Health, USA).

## Abbreviations

ITTC: Institute on Trauma and Trauma-Informed Care
NIH: National Institutes of Health
REDCap: Research Electronic Data Capture

## Data Availability

The datasets generated during and analyzed during this study are not publicly available to protect the identity of the participants, as some of the details mentioned in the qualitative interviews include information that could help identify the individual. Data are available from the corresponding author on reasonable request.
