# Supplementary material for: Developing Guidelines for Conducting Stigma Research With Transgender and Nonbinary Individuals: Protocol for Creation of a Trauma-Informed Approach to Research
Source: JMIR Res Protoc. 2025 Jan 6;14:e66800. doi: 10.2196/66800 (PMC11747536; doi:10.2196/66800)

| Multimedia Appendix 1: Interview Guide for Aims 1 and 2 by Participant Group | | |
| --- | --- | --- |
| **RQ #1 Why do people participate in or conduct research that is trans specific or on the trans community?** | | |
| **Community Members** | **Researchers** | **Mental Health Providers** |
| **To start, can you tell me a little bit about the different research studies that you’ve participated in?**  Clarifying/Deepening Probe: What were they about, what were you asked to do as a participant, what was participation like?  **1a. So one of the things we are interested in is what motivates people to participate in research. Can you tell me about why you participate in research that is on the trans community or about trans-specific issues?**  *Core Probe: What determines which research studies you participate in, and WHY? What factors help you decide whether or not to participate?*  Clarifying/Deepening Probe: Can you give me an example of a research study that you’ve been excited about, what made you excited about participating?  Clarifying/Deepening Probe: Can you give me an example of a research study you were not excited to participate in?  **1b. Thinking about the study (or studies) you’ve participated in, what are some of the benefits you’ve experienced from participating?**  Clarifying/Deepening Probe: It could be money, it could be access to medical care or other services, it might be that you learn something about yourself, or you’re giving back to the community…  **1c. What might be some deal breakers that would make you think twice about participating in a study or decide to withdraw from participation?**  Clarifying/Deepening Probe: It could be that you don’t like the way you are being treated or what you’re being asked to do, or you don’t know the real purpose of the study, or you don’t know anything about the people who are conducting the study…  **1d. What do you think motivates researchers to conduct trans-specific research?**  Clarifying/Deepening Probe: What do you think they are trying to get out of doing this research? What’s in it for them? What or who is it for? | **To start, can you tell me a little bit about the different research studies that you’ve conducted with TGNBI communities?**  Clarifying/Deepening probe: What has the focus of the research been, what methods do you use, what have been eligibility criteria?  **1d. Can you tell me about what motivated or interested you in conducting trans specific research?**  *Core Probe: What is your connection to the trans communities? How has this influenced your research?*  *Core Probe: What do you hope to accomplish in the short and long-term with your research?*  **1a. What do you think motivates trans and non-binary individuals to participate in research?**  Clarifying/Deepening Probe: What determines which research studies TGNBI participate in, and WHY? What factors do you think help these folks decide whether or not to participate?  **1b. What are some of the benefits of your trans specific research?**  *Core Probe: Benefits to the trans and non-binary communities participating in your research?*  *Core Probe: Benefits to the trans and non-binary communities more broadly?*  **1c. In your experience – not necessarily with your own studies -- what might be some deal breakers that would make a TGNB participant think twice about being in a study or decide to withdraw their participation?**  Clarifying/Deepening Probe: It could be something about the way they are treated or what they are being asked to do, or the extent to which they know the real purpose of the study, or their connection to the people who are conducting the study… | **To start, can you tell me a little bit about your clinical practice, specifically your clinical work with trans and non-binary individuals?**  Core Probe: What has the focus of your practice, what interventions do you use, what criteria do you use to decide on a treatment plan for specific clients?  Clarifying/Deepening Probe: In general, thinking about your trans and non-binary clients, why do they often come to you for therapy?  **[1d]. Can you tell me about what motivated or interested you in working with TGNB communities?**  *Core Probe: What is your connection to the trans communities? How has this influenced your practice?*  *Core Probe: What do you hope to accomplish in the short and long-term with your clinical practice?*  **1a. Thinking about the trans and non-binary communities, what do you think motivates folks to participate in research?**  Clarifying/Deepening Probe: What determines which research studies TGNBI participate in, and WHY? What factors do you think help these folks decide whether or not to participate?  **1b. What do you see as some of the benefits of trans specific research?**  *Core Probe: Benefits to you as a clinician*  *Core Probe: Benefits to the trans and non-binary communities participating in research?*  *Core Probe: Benefits to the trans and non-binary communities more broadly?*  **1c. What might be some deal breakers that would make a TGNB participant think twice about being in a study or decide to withdraw their participation?**  Clarifying/Deepening Probe: It could be something about the way they are treated or what they are being asked to do, or the extent to which they know the real purpose of the study, or their connection to the people who are conducting the study… |
| **RQ# 2: What aspects or actions within the research interaction/process are most likely to cue safety/cue threat?**   1. **Recruitment** 2. **Consent** 3. **Participation requirements** 4. **Survey Questions** 5. **Compensation (level, type, distribution schedule)** | | |
| **2a. Thinking about the research studies you’ve participated in, can you give me an example of a time you felt safe or respected during the research process?**  Clarifying/Deepening Probe: What contributed to making you feel safe and respected? It might be something about the way that you found out about the study, or what it was about, or the way that you were treated, or any incentive that you were given…  **2b. OK, now can you give me an example of something that made you feel unsafe, threatened, and uncomfortable or disrespected during the research process?**  Clarifying/Deepening Probe: It might be something about the way that you found out about the study, or what it was about, or the way that you were treated, or any incentive that you were given…  ***Now, I’m going to ask you more specifically about each part of the research process****. [NB: If the conversation above was about a specific component of the processes below, you skip that one]*  **2c. Recruitment: What stands out to you – either positive or negative – from your first interactions with a researcher, or the way you found about a study or were recruited for a study?**  *Core probe: Is there any that stands out that was [positive/negative] about these first interactions? [NB: Chose valence based on initial answer]*  **2d. Consent: What stands out to you about the informed consent process?**  *Core probe: Is there any that stands out that made you feel [comfortable/uncomfortable] about this process? [NB: Chose valence based on opposite of initial answer]*  Clarifying/Deepening probe: It might be the way the consent form was explained or presented to you, or the language that was used in the consent form or the types of things you would have to complete as a study participant or whether you were provided space to ask questions or clarify expectations to better understand what you were consenting to.  **2e. Surveys: What stands out to you when you think about surveys or questionnaires that you’ve been asked to fill out?**  Clarifying deepening probe: This might include the kinds of questions you are asked, or the language used in the survey or how often you’re asked to fill out surveys during your study participation or how long it takes to complete the survey.  *Core probe: In general, how does it make you feel to answer these types of questions?*  **2f. Research activities: What stands out to you in terms of the specific types of activities you’ve been asked to complete as a study participant?**  *Core probe: Is there any that stands out that was [positive/negative] about these activities? [NB: Chose valence based on opposite of initial answer]*  *Core probe: Is there anything about the order in which you’ve been asked to do things or the support that you were given during research participation that impacted you – either positively or negatively?*  Clarifying/Deepening probe: Sometimes you’re asked to fill out surveys, get your blood drawn, take an HIV or STI test, receive medication, participate in an interview, or focus group, track your health or emotions in a daily diary or give access to your electronic health records.  **2g. Compensation: What stands out to you in terms of the compensation or incentives you’ve received for your research participation?**  *Core probe: Is there any that stands out that was [positive/negative] about the compensation or incentives you’ve received for your research participation? [NB: Chose valence based on opposite of initial answer]*  Clarifying/Deepening probe: This might include how much you were paid, whether it was cash or gift card, how many times you were compensated or not, or how long it took to receive compensation.  **2h. Dissemination of study findings: What stands out to you about how findings from the study (or studies) you’ve participated in were shared with you?**  *Core probe: Is there any that stands out that was [positive/negative] about this process? [NB: Chose valence based on opposite of initial answer]*  Clarifying/Deepening probe: This might include how the study findings were shared with you, the language that was used to describe or talk about the findings, or who told you about the study findings or at what point in the study this happened? | **2a.** **Thinking about your research studies with TGNB communities, can you give me an example of a time when a study participant expressed that they felt safe or respected during the research process?**  Clarifying/Deepening Probe: What factors contributed to making the participant feel safe or respected? And how was this communicated to you?  **2b. Ok, now can you give me an example of a time when a study participant expressed that they felt unsafe, threatened, uncomfortable or disrespected during the research process?**  Clarifying/Deepening Probe: What factors contributed to this? And how was this communicated to you? And how did you address this?  ***Now, I’m going to ask you more specifically about your approach to conducting research and the research process****. [NB: If the conversation above was about a specific component of the processes below, you skip that one]*  **2c. Recruitment: Can you tell me a little bit about how you recruit TGNB study participants?**  Clarifying/deepening Probe: Is there anything about the way that you recruit TGNB study participants that’d different from the way you recruit other study participants?  *Core probe: How are your recruitment strategies different from other researchers’? What specific things do you try to do to make the recruitment process as safe and comfortable as possible for TGNB participants?*  **2d. Consent:** **Can you tell me a little bit about your approach to the informed consent process?**  Clarifying/deepening Probe: Is there anything about the way that you do consent for TGNB research that’s different?  *Core probe: How do you think your consent process might be different from other researchers’? What specific things do you try to do to make the consent process as safe and comfortable as possible for TGNB participants?* Clarifying/Deepening probe: This might be the language you use in the consent form, the ways in which the consent form or process is explained or presented to participants, or your approach to ensuring that potential participants feel comfortable with what they are consenting too.  **2e. Surveys: When developing surveys or questionnaires about TGNB stigma, how do you balance the need for knowledge about these issues with the concern that these topics might be upsetting or difficult for your participants?**  *Core probe: What type of harm/benefit calculation do you consider when focusing on sensitive topics?*  *Core probe: What specific things do you try to do to make the experience of taking surveys as safe and comfortable as possible for TGNB participants?*  **2f. Research Activities: How do you make decisions about the specific procedures to include in your studies?**  *Clarifying/deepening probe: Some research methods – HIV testing, providing biological samples, surveillance or monitoring – might pose emotional or even physical risks for TGNB participants. How do you make judgements about the risks and benefits of including specific procedures in your research?*  *Core probe: How do you think about the risks and benefits as it relates to possible [emotional/physical] harm [NB: Choose based on what was not included in the initial answer].*  *Core probe: Can you tell me about a time when you decided not to include something or stopped a procedure because you were worried that it would be harmful to participants?*  ***2f.2. Research activities:* Can you tell me about a time you were surprised by a reaction (either positive or negative) of a participant to a research activity, survey or interview question or procedure?**  *Core probe: In what ways have these experiences shaped your approach to conducting stigma research with TGNB communities?*  **2g. Compensation: How do you determine the type and amount of compensation or incentive you will give study participants?**  *Core probe: What specific metrics or equity factors do you apply to your decision-making process around compensation?*  **2h. Dissemination of study findings: Can you tell me a little bit about the ways in which you disseminate study findings?**  Core Probe: How do you disseminate findings to participants [if not mentioned above}  Core Probe: How do you disseminate findings to the broader TGNB community? [if not mentioned above] | **2a&b. As a clinician, can you give me some examples of actions or behaviors that may cue safety or threat to TGNB communities within the research process or interaction?**  *Core probe: What types of things would make a TGNB person feel [safe/respected or unsafe/disrespected] during the research process? [NB: Chose valence based on initial answer].*  ***Now I’m going to ask you more specifically about different parts of the research process.***  **2c. Recruitment. What types of things would cue safety or threat during the recruitment process?**  *Core probe: What types of things would make a TGNB person feel [safe/respected or unsafe/disrespected] during the research recruitment process? [NB: Chose valence based on initial answer].*  Clarifying/deepening probe: This might be the way recruitment is conducted (e.g., online vs. at events), or who is doing the recruitment, or the language used, or how the study is framed.  **2d. Informed Consent:**  **Can you give me an example of something that would cue safety or threat during the informed consent process?**  *Core probe: What types of things might make TGNB folks feel [comfortable or uncomfortable] with the consent process? [NB: Chose valence based on initial answer]*  Clarifying/Deepening probe: This might be the language used in the consent form, or how it is explained, or the types of activities associated with the study or whether there is space during the consent process to ask questions or clarify expectations to better understand what is being consented to.  **2e. Surveys: Most research on stigma and discrimination among TGNB communities is collected through online surveys or questionnaires. What do you think researchers should consider when creating survey questions that are meant to elicit responses around stigmatizing experiences or discriminatory practices?**  *Core probe: How should researchers balance the need for “knowledge generation” with the needs of TGNB communities?*  **2f. Research Activities. What do you think are some of the risks of participating in stigma related research for TGNB participants?**  *Core probe: What do you think are some of the physical risks of participating in stigma related research for TGNBI participants? [NB: If risks are generic or do not include physical risks]*    *Core probe: What do you think are some of the emotional risks of participating in stigma related research for TGNB participants? [NB: If risks are generic or do not include emotional risks]*  **2g. Compensation: What do you think is important for researchers to consider when determining the type, amount and frequency with which participants should be compensated for their study participation?**  *Core probe: What specific factors should researchers apply to their decision-making process around compensation?*  **2h. Dissemination of study findings: How can researchers ensure that their study findings are disseminated in a way that is both useful to your work and the TGNB communities?** |
| **RQ#3 (Showing participants trauma-informed care model) How do you think this model relates to the research process and what is missing?** | | |
| ***As I mentioned at the beginning of our interview, this study is about identifying strategies and developing guidelines for integrating a trauma-informed approach to conducting research with trans and non-binary communities. To ensure we’re all on the same page about what we mean when we say trauma-informed, we are going to use this infographic created by the Institute of Trauma and Trauma informed care at the University of Buffalo to help guide this part of our discussion.***  ***As we go through the infographic, I’d like you to think about how a trauma-informed approach could be applied to the research process.***  **3a. *The first part of a trauma-informed approach is realizing that trauma is widespread and impacts all areas of a person’s life and wellbeing: (read section effects of trauma from infographic).***  **3b. *The second part of a trauma-informed approach is the acknowledgement that systems and organizations may unintentionally* retraumatize *people seeking services and the individuals who work there. As you can see there are several ways re-traumatization may unintentionally happen. Read second part of infographic.***  **3c. *And the final part of this model is the integration of the five principles of a trauma-informed approach into all aspects of a system. Read final part of infographic.***  ***3d. In a minute, I’m going to ask you for more specific feedback about the 5 principles. But before, I’d like to get your initial thoughts about this model and how it may be useful both the research process and everyone involved.*** | ***As I mentioned at the beginning of our interview, this study is about identifying strategies and developing guidelines for integrating a trauma-informed approach to conducting research with trans and non-binary communities. To ensure we’re all on the same page about what we mean when we say trauma-informed, we are going to use this infographic created by the Institute of Trauma and Trauma informed care at the University of Buffalo to help guide this part of our discussion.***  ***As we go through the infographic, I’d like you to think about how a trauma-informed approach could be applied to the research process.***  **3a. *The first part of a trauma-informed approach is realizing that trauma is widespread and impacts all areas of a person’s life and wellbeing: (read section effects of trauma from infographic).***  **3b. *The second part of a trauma-informed approach is the acknowledgement that systems and organizations may unintentionally* retraumatize *people seeking services and the individuals who work there. As you can see there are several ways re-traumatization may unintentionally happen. Read second part of infographic.***  **3c. *And the final part of this model is the integration of the five principles of a trauma-informed approach into all aspects of a system. Read final part of infographic.***  ***3d. In a minute, I’m going to ask you for more specific feedback about the 5 principles. But before, I’d like to get your initial thoughts about this model and how it may be useful both the research process and everyone involved.*** | ***As I mentioned at the beginning of our interview, this study is about identifying strategies and developing guidelines for integrating a trauma-informed approach to conducting research with trans and non-binary communities. To ensure we’re all on the same page about what we mean when we say trauma-informed, we are going to use this infographic created by the Institute of Trauma and Trauma informed care at the University of Buffalo to help guide this part of our discussion.***  ***As we go through the infographic, I’d like you to think about how a trauma-informed approach could be applied to the research process.***  **3a. *The first part of a trauma-informed approach is realizing that trauma is widespread and impacts all areas of a person’s life and wellbeing: (read section effects of trauma from infographic).***  **3b. *The second part of a trauma-informed approach is the acknowledgement that systems and organizations may unintentionally* retraumatize *people seeking services and the individuals who work there. As you can see there are several ways re-traumatization may unintentionally happen. Read second part of infographic.***  **3c. *And the final part of this model is the integration of the five principles of a trauma-informed approach into all aspects of a system. Read final part of infographic.***  ***3d. In a minute, I’m going to ask you for more specific feedback about the 5 principles. But before, I’d like to get your initial thoughts about this model and how it may be useful both the research process and everyone involved.*** |
| **RQ# 4: What specific strategies have you tried or should be tried to enact a trauma-informed research practice or create a trauma-informed research environment?** | | |
| ***Building on what we’ve been talking about and based on the trauma-informed model we have shared with you, I’d like you to take a look at the “what helps?” section of the infographic again and thinking about your participation in research studies…***  **4a. What are some ways researchers can integrate these principles into their research and create a trauma-informed research environment?**  *Core probe: Let’s start with the first principle, Safety. Safety can refer to both physical and emotional safety.*  *What are some ways researchers can protect the physical or emotional safety of TGNB folks during the research process?*  *Core probe: Let’s talk about the next principle, choice. What are some ways researchers can incorporate choice into the research process?*  *Core probe: The third principle is empowerment. What are some ways that researchers can create an empowering research environment or process?*  *Core probe: What are some ways researchers can create a collaborative research environment or process?*  *Core probe: What are some ways researchers can build trust and trustworthiness?* | ***Building on what we’ve been talking about and based on the trauma-informed model we have shared with you. I’d like you to take a look at the “what helps?” section of the infographic again.***  **4a. How can we as researchers, integrate these principles into the research process to create a trauma-informed research environment?**  *Core probe: Let’s start with the first principle, Safety. Safety can refer to both physical and emotional safety.*  *What are some ways researchers can protect the physical or emotional safety of TGNB folks during the research process?*  *Core probe: Let’s talk about the next principle, choice. What are some ways researchers can incorporate choice into the research process?*  *Core probe: The third principle is empowerment. What are some ways that researchers can create an empowering research environment or process?*  *Core probe: What are some ways researchers can create a collaborative research environment or process?*  *Core probe: What are some ways researchers can build trust and trustworthiness?*  *[NB: If “include more trans people in our studies” is the strategy, probe more specifically about HOW they would do this? What does “including more trans people in the research process” really look like to them. And how do you do this from a trauma-informed framework?]* | ***Building on what we’ve been talking about and based on the trauma-informed model we have shared with you. I’d like you to take a look at the “what helps?” section of the infographic again.***  **4a. How can researchers integrate these principles into the research process to create a trauma-informed research environment?**  *Core probe: Let’s start with the first principle, Safety. Safety can refer to both physical and emotional safety.*  *What are some ways researchers can protect the physical or emotional safety of TGNB folks during the research process?*  *Core probe: Let’s talk about the next principle, choice. What are some ways researchers can incorporate choice into the research process?*  *Core probe: The third principle is empowerment. What are some ways that researchers can create an empowering research environment or process?*  *Core probe: What are some ways researchers can create a collaborative research environment or process?*  *Core probe: What are some ways researchers can build trust and trustworthiness?* |
| **RQ #5: What accountability practices can we enact in our communities to support a trauma-informed research approach?** | | |
| ***We are almost at the end of our interview. As I’ve said throughout the interview, the goal of this study is to develop a set of trauma-informed research guidelines that reduce traumatic or retraumatizing experiences for TGNB communities during the research process, but one of the struggles – is how to make this a reality. A lot of times we come to the community and ask for suggestions around best practices or guidelines, but that doesn’t mean they are actually followed or incorporated into practice.***  **5a. How can TGNB communities make sure that researchers actually follow these best practices and use a trauma-informed research approach?**  *Core probe: What criteria would you use to help you decide whether a study is safe or not safe to participate in?*  *Core probe:* What ways do folks share information about studies that are safe or not safe*?*  *Core probe: If a study did not meet your criteria, what would help you feel more empowered to refuse participation or withdraw from a study?*  *Core probe: What information do you think would help folks feel empowered to make an informed decision about whether or not to participate in a study?*    Thank you so much for all of your comments and contributions today. Are there any final ideas or thoughts that you’d like to share before we end? | ***We are almost at the end of our interview. As you know, the goal of this study is to create guidelines for research practice that reduces traumatic or retraumatizing experiences and enacts trauma-informed principles, but one of the struggles – is how to make this a reality. A lot of times there are best practices or guidelines, but that doesn’t mean they are actually followed.***  **5a. What recommendations can we make to increase the likelihood that these guidelines will actually be used by researchers?**  *Core probe: What recommendations do you have for incorporating these guidelines into the ways that RFAs or grant solicitations are written?*  *Core probe: How can we incorporate these guidelines into the grant review process?*  *Core probe: How can we incorporate them into the IRB process?*  *Core probe: How can we incorporate them into scientific publications and presentations at conferences?*  *Core probe: How can we incorporate them into the* training of mentees?  Thank you so much for all of your comments and contributions today. Are there any final ideas or thoughts that you’d like to share before we end? | ***We are almost at the end of our interview. As you know, the goal of this study is to create guidelines for research practice that reduces traumatic or retraumatizing experiences and enacts trauma-informed principles, but one of the struggles – is how to make this a reality. A lot of times there are best practices or guidelines, but that doesn’t mean they are actually followed.***  **5a. How can mental health clinicians ensure that researchers actually follow these best practices and use a trauma-informed research approach?**  *Core probe: What types of training could you provide to researchers to increase accountability?*  *Core probe:* How might you work you’re your clients to empower them to hold researchers accountable?  *Core probe: What information do you need to help folks feel empowered to make an informed decision about whether or not to participate in a study?*  Thank you so much for all of your comments and contributions today. Are there any final ideas or thoughts that you’d like to share before we end? |

Slides for Infographic to be used during RQ 3 & 4.

Adapted from the Buffalo Center for Social Research School of Social Work The Institute on Trauma and Trauma-Informed Care Simpson, R. & Green, S.A. (2014).

Adapted from: Fallot, R.D & Harris, M. (2001). Using trauma theory to design service systems: New directions for mental health services. Jossey-Bass: San Francisco, CA, Jennings, A. The Anna Institute, National Council for Community Behavioral Healthcare. Is your organization trauma-informed?


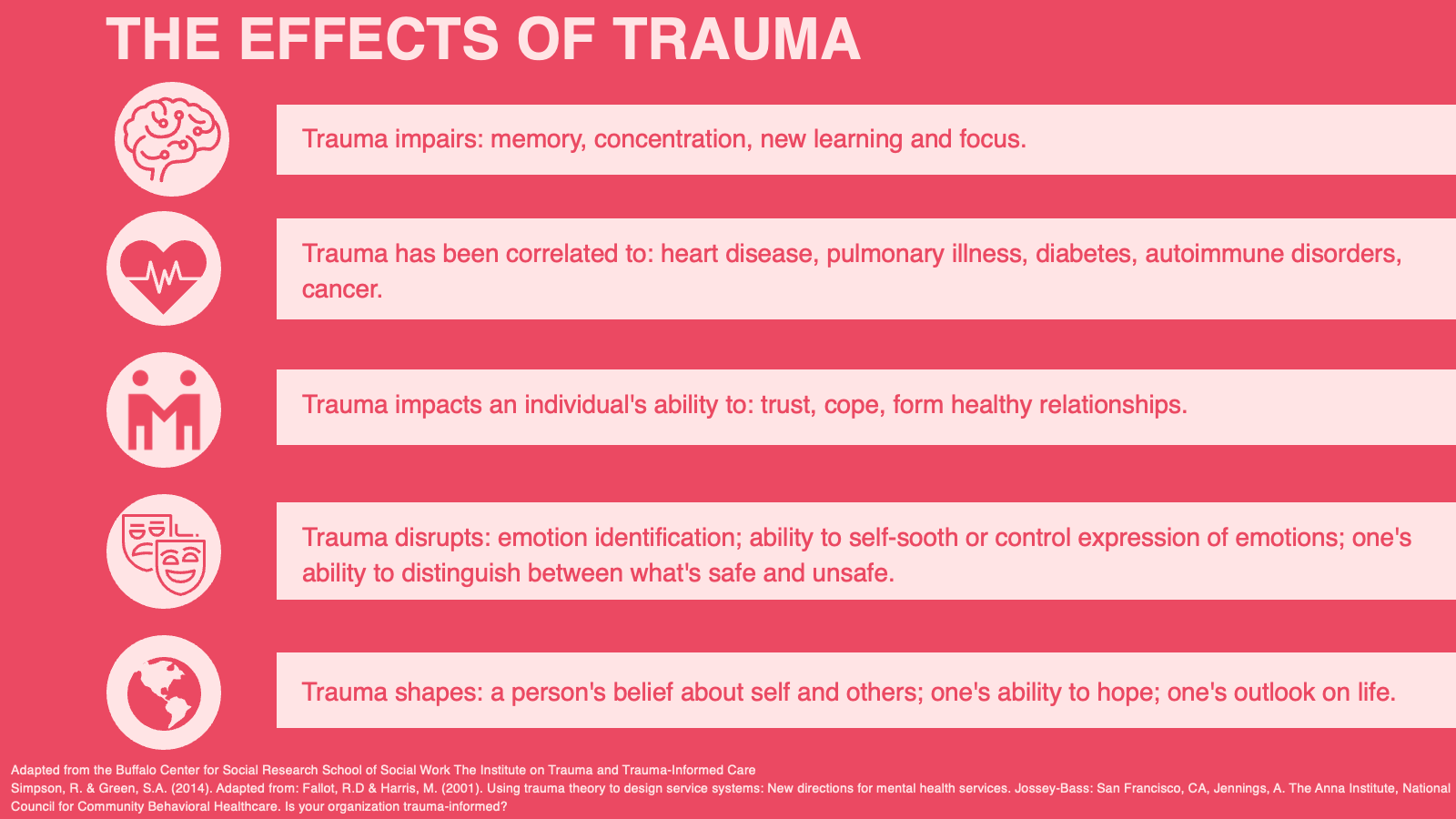


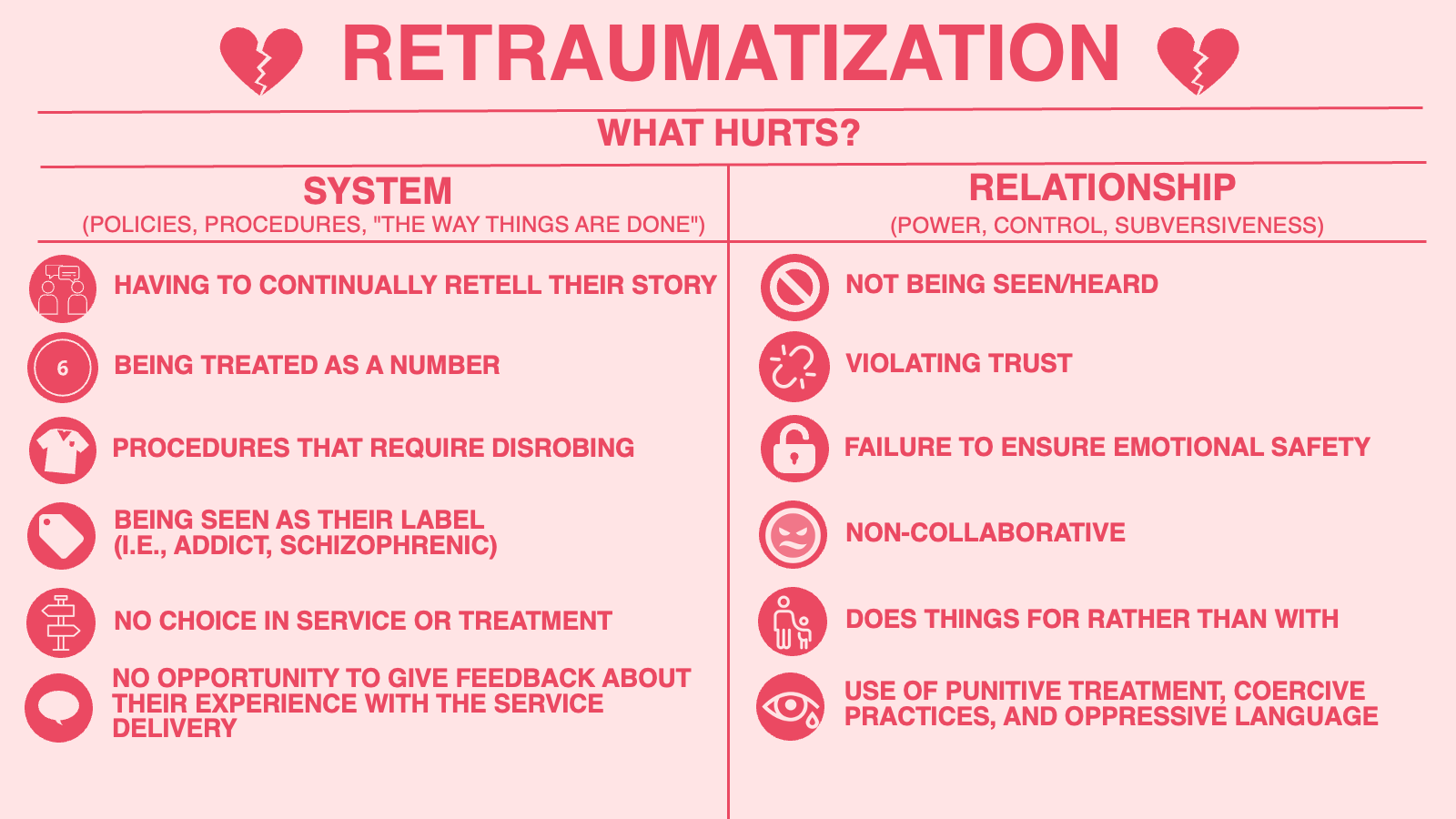


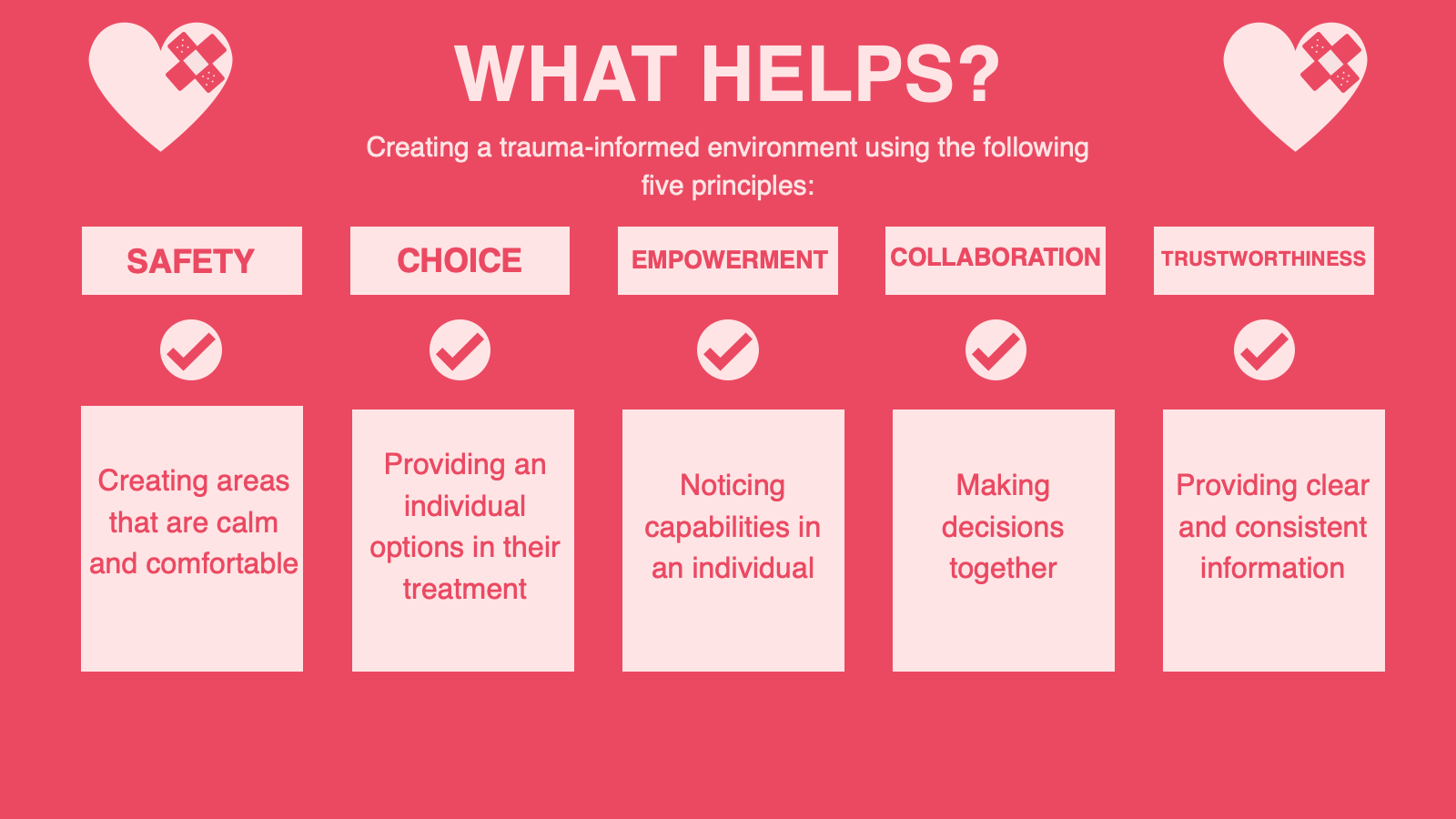

Supplement: Multimedia Appendix 1 [file resprot_v14i1e66800_app1.docx]
