# Supplementary material for: Developing Guidelines for Conducting Stigma Research With Transgender and Nonbinary Individuals: Protocol for Creation of a Trauma-Informed Approach to Research
Source: JMIR Res Protoc. 2025 Jan 6;14:e66800. doi: 10.2196/66800 (PMC11747536; doi:10.2196/66800)
Supplement: Multimedia Appendix 2 [file resprot_v14i1e66800_app2.pdf]

SUMMARY STATEMENT

PROGRAM CONTACT:
Theresa Senn
(301) 761-7852
teri.senn@nih.gov

(Privileged Communication)

Release Date: 04/01/2022
Revised Date:

Application Number: 1 R21 MH131477-01

Principal Investigators (Listed Alphabetically):

GOLUB, SARIT A (Contact)
KLEIN, AUGUSTUS

Applicant Organization: HUNTER COLLEGE

Review Group: HIBI
HIV/AIDS Intra- and Inter-personal Determinants and Behavioral Interventions Study Section
AIDS

Meeting Date: 03/10/2022
Council: MAY 2022
Requested Start: 07/01/2022

RFA/PA: PA21-235
PCC: 9A-ASGP

Project Title: Trauma-Informed Investigation of TGNBI Experiences in HIV Stigma Research

SRG Action: Impact Score:24 Percentile:9 +
Next Steps: Visit https://grants.nih.gov/grants/next_steps.htm
Human Subjects: X2-Human subjects involved - Exemption #2 designated
Animal Subjects: 10-No live vertebrate animals involved for competing appl.
Gender: 1A-Both genders, scientifically acceptable
Minority: 1A-Minorities and non-minorities, scientifically acceptable
Age: 3A-No children included, scientifically acceptable

Project Year	Direct Costs Requested	Estimated Total Cost
1	175,000	273,000
2	100,000	156,000
TOTAL	275,000	429,000

ADMINISTRATIVE BUDGET NOTE: The budget shown is the requested budget and has not been adjusted to reflect any recommendations made by reviewers. If an award is planned, the costs will be calculated by Institute grants management staff based on the recommendations outlined below in the COMMITTEE BUDGET RECOMMENDATIONS section.

1R21MH131477-01 Golub, Sarit

RESUME AND SUMMARY OF DISCUSSION: This application seeks to develop a set of recommendations for investigations that are conducting HIV-related stigma and discrimination research with transgender and non-binary individuals (TGNBI). First, 30 in-depth interviews will be conducted with TGNBI that will be stratified by gender and identity status, with most participants being under 30 and people of color. Second, 15 in-depth interviews will be conducted with investigators that conducting HIV-related stigma and discrimination research with TGNBI. Another 15 interviews will be conducted with mental health professionals that provide care to TGNBI. Finally, a modified Delphi technique will be used with a panel made up of TGNBI, investigators and mental health providers to identify practical recommendations for the person-centered, trauma-informed conducting of HIV-related stigma research with TGNBI. The committee found this to be a highly innovative application and that the study team is experienced and very strong. It was suggested that the team might benefit from the addition of an ethicist. The committee noted a few minor weaknesses in the proposed study, but it was also felt that the potential impact of the proposed practical recommendations for investigators conducting HIV-related research with TGNBI could be high.

DESCRIPTION (provided by applicant): Transgender and non-binary individuals (TGNBI) have received increasing attention within HIV research, with studies documenting the pervasive role stigma plays in creating and sustaining health inequities. However, the proliferation of HIV stigma research with this population has also raised concerns about research practices that may unintentionally stigmatize or re-traumatize the very communities they are designed to benefit. Conducting stigma research is critical for generating accurate information about HIV epidemiology, risk and protective factors, and intervention strategies for TGNBI. Yet little, if any, research has directly examined the experiences of TGBNI when participating in these studies, or identified specific research practices (e.g., recruitment materials/study framing, choice of specific survey measures, data collection protocols, researcher behaviors) that may influence study participation, retention, and data quality. Equally important, research has not adequately examined the potential for unintended harm due to emotional distress experienced by participating in such research and what specific strategies might mitigate against potential distressful research experiences. To make meaningful strides in stigma research, it is imperative to develop a set of empirically-grounded practical recommendations for conducting this research with TGNBI in a manner that is person-centered, trauma informed, and actively de-stigmatizing. This area of inquiry is timely and particularly relevant given the NIH's commitment to building investigators' capacity for conducting intersectional multidisciplinary stigma research. In October 2021, the NIH released a Stigma and Discrimination Research Toolkit, which is an important contribution to health-related stigma research and represents a foundational shift towards addressing stigma through a health equity framework. However, one of the critical gaps in this toolkit is the lack of research examining experiences of stigma within the research context and identifying strategies for improving data quality and reducing unintentional harm in study recruitment, methodology, and/or implementation. As such, the specific aims of this exploratory R21 project are to: (1) Conduct in-depth interviews with TGNBI, stratified by gender identity and HIV status, to better understand how TGNBI understand and experience participation in HIV related stigma and discrimination research; (2) Conduct in-depth interviews with both investigators who conduct HIV-related stigma and discrimination research with TGNBI and mental health professionals who provide care to TGNBI to better understand perceptions of and experiences with conducting stigma and discrimination research with TGNBI, and compile existing strategies for mitigating harm; and (3) Utilize a modified Delhi technique to develop a set of empirically-informed guidelines for conducting HIV stigma research with TGNBI. The present study will be the first (to our knowledge) to develop evidence-based research guidelines to: a) increase researchers' capacity to recruit and retain TGNBI in HIV-related stigma research, b) enhance the quality of data collected; and c) reduce unintentional harm in HIV stigma research methodology.

PUBLIC HEALTH RELEVANCE: This project is designed to address critical gaps in research on HIV stigma among transgender and non-binary individuals (TGNBI) by examining experiences of stigma within the research context and identifying strategies for improving data quality and reducing unintentional harm in study recruitment, methodology, and/or implementation. This project is poised to have an immediate, significant, and sustained impact on HIV stigma research and has the potential to influence research relevant to other high-priority populations.

CRITIQUE 1

Significance: 4
Investigator(s): 3
Innovation: 2
Approach: 4
Environment: 3

Overall Impact: This R21 application, "Trauma-Informed Investigation of TGNBI Experiences in HIV Stigma Research," proposes to create a set of evidence-based HIV stigma research guidelines specific to transgender and non-binary individuals (TGNBI) by conducting qualitative interviews with TGBNI and with stigma researchers and mental health professionals. The application is highly innovative and is significant in its attempt to explore stigma and re-victimization endured by this population within the research context. The proposed research is compelled by historical experiences of exploitation by TGNBI within the context of research studies, and by extant instruments assessing internalized stigma. Of particular note is the attention paid to unintentional harm experienced by TGBNI participating in HIV-related research: the scientific premise is that HIV stigma research can either recapitulate oppression or disrupt oppression. Potential for impact is moderately reduced by the diffuse nature of its key deliverable (empirically-informed guidelines) and understated plans to effectively disseminate this deliverable in a way that can optimally achieve uptake by the HIV stigma research community; a research team that overlaps in expertise and lacks bioethical expertise and psychometric expertise; and lack of attention to development of new instrumentation that could more saliently assess experiences of intersectional stigma (including HIV-related stigma) faced by TGNBI.

1. Significance:

Strengths

- The application makes a solid argument that existing stigma instrumentation (especially internalized measures) have the potential to cause unintended emotional distress, especially to TGBNI research participants.
- Linking patient-centered, trauma-informed models of care to intersectional stigma research approaches with highly marginalized populations such as TGBNI of color is a significant conceptual development and is nicely described.
- The application engages with newly emerging research on intersectional stigma and the development of guidelines and strategies that are recent byproducts of the NIH Working Group on HIV and Intersectional Stigma. Of particular note here is the scarcity of extant stigma measures created with (and for) TGBNI communities of color.

Weaknesses

- Some assertions that are foundational to the proposed research are unsupported/unsourced, such as the statement that participation in research could be stigmatizing and could thus negatively impact future HIV prevention and care involvement by participants.
- There is little discussion about the potential impact a set of empirically-informed guidelines such as proposed in Aim 3 (key deliverable) would have on the field of HIV stigma research, or how this impact could be maximized.

2. Investigator(s):

Strengths

- The team is led by an experienced investigator (PI: Golub) in HIV prevention research with TGBNI populations.
- The investigative team is reflective of the TGNBI community it proposes to research, which will likely increase the saliency of the research conducted.

Weaknesses

- The research team could be strengthened by the addition of a bioethics expert and by the addition of a psychometrician with experience in multidimensional, intersectional stigma constructs.
- There appears to be overlap in expertise among the research team, which is primarily composed of experts in psychology.

3. Innovation:

Strengths

- Proposes to qualitatively explore relationships between participation in HIV stigma research and unintended negative consequences in a population that experiences high levels of marginalization, representing a conceptual innovation.
- Leverages patient-centered, trauma-informed framework and applies it to research participation issues.
- Proposes to convene a diverse array of stakeholders (researchers, mental health professionals, TGBNI community members) on equal footing to develop recommendations for future work in this arena (Aim 3).

Weaknesses

- No substantive methodological (e.g., analytic; instrument-based) innovations are offered.

4. Approach:

Strengths

- The description of the data collection and analytic methods are sound and appear rigorous and sufficient to assess the study’s proposed aims. Particularly notable is the attention given to implementing the Delphi method, in this case using a three-round approach in order to generate a set of recommendations that achieves consensus.

- Preliminary studies informing the approach provide strong rationale for addressing unintended consequences of instruments with poor salience and/or highly negative valence (though it is difficult to infer the exact measures that quoted participants are responding to here).
- Preliminary studies underscore the value for participant completion and retention in research that comes as a result of intentionally designing and refining instruments to optimize salience to marginalized populations.
- The theoretical framework is well-founded on minority stress theory and trauma-informed care.
- Multimodal recruitment strategy (health centers, social media, word of mouth) is likely to succeed in achieving the proposed sample size.

Weaknesses

- More justification related to the proposed sample size for qualitative aim 1 (n=30, stratified by gender identity and HIV status, with 70% people of color and >40% under the age of 30) and aim 2 (15 mental health professionals, 15 HIV stigma researchers) would strengthen the analytic plan. It is possible though somewhat unlikely, saturation will be achieved, especially after stratification in Aim 1.
- It is unclear how the key deliverable (a set of empirically-informed guidelines for HIV stigma research with TGNBI populations) will optimally impact the HIV stigma research community. What dissemination approaches and key stakeholders could maximize adherence by HIV stigma researchers of the guidelines that will be promulgated? What barriers and challenges do the applicants foresee in eventual adoption of such guidelines by the research community, and how might these challenges be confronted?
- Given that the measures described as potentially harmful to TGNBI participants are related to internalized stigma scales (internalized HIV stigma; internalized transphobia), and that such measures have generally been adapted from existing scales developed primarily for use among gay men, there seems to be a clear and pressing need to design new measures in close collaboration with TGNBI communities, particularly TGNBI of color, and to then test these measures for salience, construct validity, and reliability. Perhaps these recommendations will come out in Aim 3 as part of the guidelines, but it is perhaps a missed opportunity here to not attend to this proposal's potential to help generate less harmful and more salient measures related to internalized stigma across the intersections of gender identity, HIV status, and minority race/ethnicity.
- More attention could be given to intersectionality and intersectional stigma and how they intertwine with minority stress theory and trauma-informed care here.
- Some attention to potential limitations and consideration of alternate strategies could strengthen this proposal.

5. Environment:

Strengths

- The research environment at Hunter College, Callen-Lorde, and Saybrook University is sufficient to support this work.

Weaknesses

- Formal arrangements with the 4 other CBOs who will help recruit participants could strengthen this application. Additional letters of support from these organizations have not been provided.

Study Timeline:

Strengths

- The timeline is appropriate.

Weaknesses

- None noted by reviewer.

Protections for Human Subjects:

Acceptable Risks and/or Adequate Protections

Data and Safety Monitoring Plan (Applicable for Clinical Trials Only):

Not Applicable (No Clinical Trials)

Inclusion Plans:

- Sex/Gender: Distribution justified scientifically
- Race/Ethnicity: Distribution justified scientifically
- For NIH-Defined Phase III trials, Plans for valid design and analysis: Not applicable
- Inclusion/Exclusion Based on Age: Distribution justified scientifically

Vertebrate Animals:

Not Applicable (No Vertebrate Animals)

Biohazards:

Not Applicable (No Biohazards)

Resource Sharing Plans:

Acceptable

Budget and Period of Support:

Recommend as Requested

CRITIQUE 2

Significance: 2
Investigator(s): 2
Innovation: 2
Approach: 3
Environment: 1

Overall Impact: Transgender and non-binary individuals (TGNBI) have received increasing attention within HIV research, with studies documenting the pervasive role stigma plays in creating and

sustaining health inequities. This exploratory R21 project aims to: (1) Conduct in-depth interviews with TGNBI, stratified by gender identity and HIV status, to better understand how TGNBI understand and experience participation in HIV related stigma and discrimination research; (2) Conduct in-depth interviews with both investigators who conduct HIV-related stigma and discrimination research with TGNBI and mental health professionals who provide care to TGNBI to better understand perceptions of and experiences with conducting stigma and discrimination research with TGNBI, and compile existing strategies for mitigating harm; and (3) Utilize a modified Delphi technique to develop a set of empirically-informed guidelines for conducting HIV stigma research with TGNBI. The study will yield practical findings that are of use to TGNBI as well as individuals who engage in research with this population. Relatively few and minor weaknesses can be found in this otherwise very compelling proposal.

1. Significance:

Strengths

- The measurement of stigma as part of HIV-related research with transgender and non-binary individuals (TGNBI) is significant. There is concern that specific items within commonly used measures may unintentionally recreate or activate stigma.
- There are no evidence-based guidelines for person-centered, trauma-informed and actively de-stigmatizing implementation of HIV stigma research.
- It is imperative to develop a set of empirically-grounded practical recommendations for conducting this research with TGNBI in a manner that is person-centered, trauma informed, and actively de-stigmatizing.
- Preliminary evidence from the research team provides empirical support for the proposed study.
- This would represent the first NIH funded study that specifically examines the impact of HIV stigma research methods on the experience of study participation among TGNBI.

Weaknesses

- None noted.

2. Investigator(s):

Strengths

- MPI Dr. Golub has several decades of experience collaborating with community-based organizations that provide HIV prevention and care services to transgender and non-binary individuals (TGNBI) as well as other sexual and gender minorities (SGM).
- MPI Dr. Klein is a recognized expert in transgender health research and has been a leader in and advocate for representation of transgender researchers and community members in study design, implementation, and dissemination.
- Co-I Dr. Berke is a licensed Clinical Psychologist with clinical expertise in the assessment and psychological treatment of trauma and stress-related disorders and in the provision of gender-affirming clinical programming and psychological care practices tailored to the needs of diverse TGBN clients and communities.
- Strong research coordinators and collaborators round out the study team.

Weaknesses

- None noted

3. Innovation:

Strengths

- The study would represent the first NIH funded study that specifically examines the impact of HIV stigma research methods on the experience of study participation among TGNBI.
- The study has significant implications for both clinical and research outcomes.
- The research team has a commitment to collaboration with and representation of transgender individuals in their research.

Weaknesses

- None noted.

4. Approach:

Strengths

- Findings from preliminary studies have guided the development of this project.
- The proposed study is theoretically grounded in minority stress and trauma-informed approaches.
- Investigators will conduct a series of in-depth semi-structured qualitative interviews with thirty (N=30) transgender and non-binary individuals (TGNBI). Participants will be stratified by gender identity and HIV status.
- Description of modified Delphi process is sufficiently detailed.

Weaknesses

- Data analysis with grounded theory is only discussed at a surface level.
- It is unclear whether the sample sizes of interviews with researchers and mental health professionals will be sufficient to yield clear findings, particularly when stratified by career stage.

5. Environment:

Strengths

- Hunter College has the resources to carry out the proposed study.

Weaknesses

- None noted.

Study Timeline:

Strengths

- Study timeline is sufficiently described.

Weaknesses

- None noted by reviewer.

Protections for Human Subjects:

Acceptable Risks and/or Adequate Protections
Data and Safety Monitoring Plan (Applicable for Clinical Trials Only):
Acceptable

Inclusion Plans:

- Sex/Gender: Distribution justified scientifically
- Race/Ethnicity: Distribution justified scientifically
- For NIH-Defined Phase III trials, Plans for valid design and analysis: Not applicable
- Inclusion/Exclusion Based on Age: Distribution justified scientifically

Vertebrate Animals:
Not Applicable (No Vertebrate Animals)

Biohazards:
Not Applicable (No Biohazards)

Resource Sharing Plans:
Not Applicable (No Relevant Resources)

Budget and Period of Support:
Recommend as Requested

CRITIQUE 3

Significance: 1
Investigator(s): 2
Innovation: 2
Approach: 3
Environment: 1

Overall Impact: This application from an experienced research team seeks to understand the experience of participating in stigma-related research among trans and non-binary participants, as well as researchers and mental health practitioners, and develop a set of evidence-based guidelines using the Delphi technique. The research team is experienced in stigma research with trans and non-binary individuals, and includes trans identified team members. Two rounds of qualitative interviews, one with trans and non-binary individuals who have participated in prior stigma-related research and the second round with researchers and mental health workers who study stigma or treat trauma among trans and non-binary individuals. There is a plan for how these data will be used together to develop trauma-informed and person-centered guidelines for research, using the language of participants as much as possible, that will then be used for the Delphi technique. Three rounds of ratings by trans and non-binary individuals, researchers, and mental health providers will seek consensus on guidelines. Overall

the plan is sound, though some details are vague or overlooked. It might benefit the team to include a bioethicist or institutional IRB expert given the research administration implications of this work.

1. Significance:

Strengths

- Improving the quality of stigma related research with trans and non-binary people by developing evidence-based research guidelines and tools is an important goal
- Understanding potential unanticipated and unintended harm resulting from participating in research is also important

Weaknesses

- None noted

2. Investigator(s):

Strengths

- MPI team headed by Drs. Golub and Klein, and includes trans-identified investigators
- Dr. Golub is a Social Psychologist and an experienced researcher with expertise studying stigma and the health impacts of stigma on sexual and gender minority populations, including trans and non-binary individuals and qualitative research methods
- Dr. Klein has a background in Social Work and has training and experience in health disparities research and implementation science. Dr. Klein has a great deal of experience working with community-based organizations that serve people living with and at risk for HIV, and has research experience with trans and non-binary individuals
- Dr. Berke is a Clinical Psychologist with both clinical and research expertise on traumatic stress and gender-affirming therapy for trans and non-binary individuals

Weaknesses

- While this team is highly experienced and has tremendous clinical and community experience, having someone with more of an institutional focus—perhaps a bioethicist or IRB specialist—might add a meaningful dimension to the team

3. Innovation:

Strengths

- Examining the impact of participating in stigma-related research is innovative
- Seeking to establish evidence-based guideline for stigma research with trans and non-binary individuals is important and needed

Weaknesses

- The methodology is not particularly innovative

4. Approach:

Strengths

- Builds on work by the study team that identified positive and negative implications of research participation among trans participants
- Integrates Minority Stress Theory with trauma informed care that is person centered and strengths based
- Aim 1 will recruit 30 transgender and non-binary participants, stratified by gender identity and HIV status, with over 40% 18 to 29 years of age and 70% people of color. Inclusion criteria require that individuals have participated in prior research studies
- Interviews will focus on the experience of participating in stigma-related research
- Aim 2 will focus on qualitative interviews with 15 researchers who have conducted HIV stigma and discrimination research with trans and non-binary individuals and 15 mental health providers who serve these individuals
- Interviews will focus on the experience of conducting stigma-related research and measuring stigma using person-centered, trauma-informed language
- Overall, analysis plan is reasonable, and a clear description of how data from Aims 1 and 2 will be used to develop potential survey items to be used in Aim 3. Items will use language used by participants in interviews and will represent key themes identified in the data
- Aim 3 will conduct a three round survey using a Delphi technique to obtain feedback on the items (I assume items referred to in the application equate with the guidelines the study aims to create). The Delphi panel will include 75 participants, 25 trans and non-binary individuals, 25 researchers, and 25 mental health providers

Weaknesses

- Would it be beneficial to include medical providers, not just mental health providers—particularly medical providers that serve trans individuals?
- Will interviews and approach in Aims 1 and 2 be open to revision based on ongoing data collection? Will constant comparison and an iterative process be used?
- Will member checks be conducted to make sure data analysis is reflecting the experience of the participants?
- Application notes that a document will be prepared based on results to be widely disseminated—but does not explain how it will be disseminated. One important avenue would be a structural intervention or training/operating guidelines for IRBs and research training programs
- This is a starting point for this line of research, as research guidelines are probably needed beyond the construct of stigma. Also, this is needed beyond New York City—given what is happening in Texas and Florida, for example, one can imagine that capturing the perspectives of LGBTQ+ individuals in the South would be valuable

5. Environment:

Strengths

- Hunter College of the City University of New York is an excellent setting to conduct the proposed research
- Support from both the ERC-CFAR and the HIV Center at New York Psychiatric Institute and Columbia is an additional strength

Weaknesses

- None noted

Protections for Human Subjects:

Acceptable Risks and/or Adequate Protections

- Risks are minor and include discomfort in discussing personal information and loss of confidentiality. Data security appears to be adequate and staff will be trained to identify and address discomfort and distress

Data and Safety Monitoring Plan (Applicable for Clinical Trials Only):

Not Applicable (No Clinical Trials)

Inclusion Plans:

- Sex/Gender: Distribution justified scientifically
- Race/Ethnicity: Distribution justified scientifically
- For NIH-Defined Phase III trials, Plans for valid design and analysis: Not applicable
- Inclusion/Exclusion Based on Age: Distribution justified scientifically
- Study will recruit a broad range of race/ethnicity and sexual/gender identify and ages--seeks a diverse sample, including trans and non-binary individuals, researchers and mental health providers

Vertebrate Animals:

Not Applicable (No Vertebrate Animals)

Biohazards:

Not Applicable (No Biohazards)

Resource Sharing Plans:

Not Applicable (No Relevant Resources)

Authentication of Key Biological and/or Chemical Resources

Not Applicable (No Relevant Resources)

Budget and Period of Support:

Recommend as Requested

THE FOLLOWING SECTIONS WERE PREPARED BY THE SCIENTIFIC REVIEW OFFICER TO SUMMARIZE THE OUTCOME OF DISCUSSIONS OF THE REVIEW COMMITTEE, OR REVIEWERS' WRITTEN CRITIQUES, ON THE FOLLOWING ISSUES:

PROTECTION OF HUMAN SUBJECTS: ACCEPTABLE

INCLUSION OF WOMEN PLAN: ACCEPTABLE

INCLUSION OF MINORITIES PLAN: ACCEPTABLE

INCLUSION ACROSS THE LIFESPAN: ACCEPTABLE

COMMITTEE BUDGET RECOMMENDATIONS: The budget was recommended as requested.

Footnotes for 1 R21 MH131477-01; PI Name: Golub, Sarit A

+ Derived from the range of percentile values calculated for the study section that reviewed this application.

NIH has modified its policy regarding the receipt of resubmissions (amended applications).See Guide Notice NOT-OD-18-197 at https://grants.nih.gov/grants/guide/notice-files/NOT-OD-18-197.html. The impact/priority score is calculated after discussion of an application by averaging the overall scores (1-9) given by all voting reviewers on the committee and multiplying by 10. The criterion scores are submitted prior to the meeting by the individual reviewers assigned to an application, and are not discussed specifically at the review meeting or calculated into the overall impact score. Some applications also receive a percentile ranking. For details on the review process, see http://grants.nih.gov/grants/peer_review_process.htm#scoring.

MEETING ROSTER

HIV/AIDS Intra- and Inter-personal Determinants and Behavioral Interventions Study Section
Risk, Prevention and Health Behavior Integrated Review Group
CENTER FOR SCIENTIFIC REVIEW
HIBI
03/10/2022 - 03/11/2022

Notice of NIH Policy to All Applicants: Meeting rosters are provided for information purposes only. Applicant investigators and institutional officials must not communicate directly with study section members about an application before or after the review. Failure to observe this policy will create a serious breach of integrity in the peer review process, and may lead to actions outlined in NOT-OD-14-073 at https://grants.nih.gov/grants/guide/notice-files/NOT-OD-14-073.html, NOT-OD-15-106 at https://grants.nih.gov/grants/guide/notice-files/NOT-OD-15-106.html, and NOT-OD-18-115 at https://grants.nih.gov/grants/guide/notice-files/NOT-OD-18-115.html, including removal of the application from immediate review.

CHAIRPERSON(S)

KIPKE, MICHELE D, PHD
PROFESSOR
DEPARTMENTS OF PEDIATRICS
AND PREVENTIVE MEDICINE
KECK SCHOOL OF MEDICINE
UNIVERSITY OF SOUTHERN CALIFORNIA
LOS ANGELES, CA 90028

MEMBERS

BUTLER, LISA MICHELLE, PHD
ASSOCIATE RESEARCH PROFESSOR
INSTITUTE FOR COLLABORATION ON HEALTH,
INTERVENTION, AND POLICY
UNIVERSITY OF CONNECTICUT
STORRS, CT 06269

COMULADA, WARREN SCOTT, DRPH
ASSOCIATE PROFESSOR
DEPARTMENT OF PSYCHIATRY
AND BIOBEHAVIORAL SCIENCES
SCHOOL OF PUBLIC HEALTH
UNIVERSITY OF CALIFORNIA, LOS ANGELES
LOS ANGELES, CA 90024

DODGE, BRIAN MARK, PHD
PROFESSOR
DEPARTMENT OF APPLIED HEALTH SCIENCE
INDIANA UNIVERSITY SCHOOL OF PUBLIC HEALTH
BLOOMINGTON, IN 47405

FRIEDMAN, MACKEY R, PHD *
ASSISTANT PROFESSOR
DEPARTMENT OF INFECTIOUS DISEASES AND
MICROBIOLOGY
UNIVERSITY OF PITTSBURGH
PITTSBURGH, PA 15213

GILBERTSON, ADAM L, PHD *
ASSOCIATE RESEARCH SCIENTIST
PACIFIC INSTITUTE FOR RESEARCH AND EVALUATION
CHAPEL HILL, NC 27514

GOLUB, JONATHAN E, MPH, PHD *
ASSOCIATE PROFESSOR
CENTER FOR TUBERCULOSIS RESEARCH
SCHOOL OF MEDICINE
JOHNS HOPKINS UNIVERSITY
BALTIMORE, MD 21231

GRAHAM, SUSAN MARIE, MD, PHD
PROFESSOR
DEPARTMENTS OF MEDICINE AND GLOBAL HEALTH
SCHOOL OF MEDICINE
UNIVERSITY OF WASHINGTON
SEATTLE, WA 98104

GROV, CHRISTIAN, PHD
PROFESSOR AND CHAIR
DEPARTMENT OF COMMUNITY HEALTH
AND SOCIAL SCIENCES
SCHOOL OF PUBLIC HEALTH AND HEALTH POLICY
CITY UNIVERSITY OF NEW YORK
NEW YORK, NY 10027

HAMPANDA, KAREN MARIE, MPH, PHD *
ASSISTANT PROFESSOR
DEPARTMENT OF OBSTETRICS AND GYNECOLOGY
UNIVERSITY OF COLORADO ANSCHUTZ MEDICAL CAMPUS
DENVER, CO 80045

HANSEN, NATHAN B, PHD
DEPARTMENT HEAD AND PROFESSOR
DEPARTMENT OF HEALTH PROMOTION AND BEHAVIOR
COLLEGE OF PUBLIC HEALTH
UNIVERSITY OF GEORGIA
ATHENS, GA 30602

HEADS, ANGELA, PHD *
ASSOCIATE PROFESSOR
DEPARTMENT OF PSYCHIATRY AND BEHAVIORAL SCIENCES
UNIVERSITY OF TEXAS HEALTH SCIENCES CENTER AT HOUSTON
HOUSTON, TX 77054

HORVATH, KEITH JOSEPH, PHD
ASSOCIATE PROFESSOR
DEPARTMENT OF CLINICAL PSYCHOLOGY
SAN DIEGO STATE UNIVERSITY
SAN DIEGO, CA 92120

IWELUNMOR, JULIET, PHD
ASSOCIATE PROFESSOR
DEPARTMENT OF BEHAVIORAL SCIENCE AND HEALTH EDUCATION
COLLEGE FOR PUBLIC HEALTH AND SOCIAL JUSTICE
ST. LOUIS UNIVERSITY
ST. LOUIS, MO 63104

KIM, MARIA HYOUN, MD *
ASSOCIATE PROFESSOR
DEPARTMENT OF PEDIATRICS
BAYLOR COLLEGE OF MEDICINE
HOUSTON, TX 77030

LOVEJOY, TRAVIS IAN, PHD
ASSOCIATE PROFESSOR
DEPARTMENT OF PSYCHIATRY
SCHOOL OF MEDICINE
OREGON HEALTH AND SCIENCE UNIVERSITY
PORTLAND, OR 97239

MACDONELL, KAREN KOLMODIN, PHD *
ASSOCIATE PROFESSOR
DEPARTMENT OF FAMILY MEDICINE AND PUBLIC HEALTH SCIENCES
SCHOOL OF MEDICINE
WAYNE STATE UNIVERSITY
DETROIT, MI 48202

MACKESY-AMITI, MARY ELLEN, PHD *
ASSOCIATE PROFESSOR
DIVISION OF EPIDEMIOLOGY AND BIOSTATISTICS
SCHOOL OF PUBLIC HEALTH
UNIVERSITY OF ILLINOIS, CHICAGO
CHICAGO, IL 60453

PATEL, VIRAJ V, MPH, MD *
ASSOCIATE PROFESSOR OF MEDICINE
DEPARTMENT OF MEDICINE
ALBERT EINSTEIN COLLEGE OF MEDICINE
BRONX, NY 10461

RAMSEY, SUSAN E, PHD
ASSOCIATE PROFESSOR
DIVISION OF GENERAL INTERNAL MEDICINE
RHODE ISLAND HOSPITAL
BROWN UNIVERSITY
PROVIDENCE, RI 02903

ROTH, ALEXIS MARIE, MPH, PHD *
ASSOCIATE PROFESSOR
DEPARTMENT OF COMMUNITY HEALTH & PREVENTION
DORNSIFE SCHOOL OF PUBLIC HEALTH
DREXEL UNIVERSITY
PHILADELPHIA, PA 19104

SAFREN, STEVEN A, PHD
PROFESSOR
DEPARTMENT OF PSYCHOLOGY
COLLEGE OF ARTS AND SCIENCES
UNIVERSITY OF MIAMI
CORAL GABLES, FL 33124

SSEWAMALA, FRED M, PHD
PROFESSOR
INSTITUTE FOR PUBLIC HEALTH
BROWN SCHOOL
WASHINGTON UNIVERSITY
ST. LOUIS, MO 63130

STATON, MICHELE, PHD *
PROFESSOR
DEPARTMENT OF BEHAVIORAL SCIENCES
COLLEGE OF MEDICINE
UNIVERSITY OF KENTUCKY
LEXINGTON, KY 40536

STOCKMAN, JAMILA KINSHASA, PHD
PROFESSOR
DIVISION OF GLOBAL PUBLIC HEALTH
DEPARTMENT OF MEDICINE
SCHOOL OF MEDICINE
UNIVERSITY OF CALIFORNIA, SAN DIEGO
LA JOLLA, CA 92093

SULLIVAN, PATRICK SEAN, PHD
PROFESSOR
DEPARTMENT OF EPIDEMIOLOGY
ROLLINS SCHOOL OF PUBLIC HEALTH
EMORY UNIVERSITY
ATLANTA, GA 30322

TANNER, AMANDA E, MPH, PHD *
ASSOCIATE PROFESSOR
DEPARTMENT OF PUBLIC HEALTH EDUCATION
SCHOOL OF HEALTH AND HUMAN SCIENCES
UNIVERSITY OF NORTH CAROLINA GREENSBORO
GREENSBORO, NC 27402

THAMES, APRIL D, PHD
ASSOCIATE PROFESSOR
DEPARTMENT OF PSYCHIATRY AND BEHAVIORAL SCIENCES
UNIVERSITY OF CALIFORNIA, LOS ANGELES
LOS ANGELES, CA 90095

TURAN, JANET M, PHD
PROFESSOR
DEPARTMENT OF HEALTH CARE ORGANIZATION AND POLICY
SCHOOL OF PUBLIC HEALTH
UNIVERSITY OF ALABAMA AT BIRMINGHAM
BIRMINGHAM, AL 35294

WEBEL, ALLISON R, PHD
PROFESSOR
SCHOOL OF NURSING
UNIVERSITY OF WASHINGTON
SEATTLE, WA 98195

WILTON, LEO, PHD
PROFESSOR
DEPARTMENT OF HUMAN DEVELOPMENT
COLLEGE OF COMMUNITY AND PUBLIC AFFAIRS
BINGHAMTON UNIVERSITY
BINGHAMTON, NY 13902

WINDSOR, LILIANE CAMBRAIA, PHD
ASSOCIATE PROFESSOR
SCHOOL OF SOCIAL WORK
THE UNIVERSITY OF ILLINOIS AT URBANA-CHAMPAIGN
URBANA, IL 61801

SCIENTIFIC REVIEW OFFICER

RUBERT, MARK P, PHD
SCIENTIFIC REVIEW OFFICER
CENTER FOR SCIENTIFIC REVIEW
NATIONAL INSTITUTES OF HEALTH
BETHESDA, MD 20892

EXTRAMURAL SUPPORT ASSISTANT

CAMBRELEN, AMY ANGELA
EXTRAMURAL SUPPORT ASSISTANT
CENTER FOR SCIENTIFIC REVIEW
NATIONAL INSTITUTE OF HEALTH
BETHESDA, MD 20892

* Temporary Member. For grant applications, temporary members may participate in the entire meeting or may review only selected applications as needed.

Consultants are required to absent themselves from the room during the review of any application if their presence would constitute or appear to constitute a conflict of interest.
